# Alterations on peripheral B cell subsets following an acute uncomplicated clinical malaria infection in children

**DOI:** 10.1186/1475-2875-7-238

**Published:** 2008-11-18

**Authors:** Amolo S Asito, Ann M Moormann, Chelimo Kiprotich, Zipporah W Ng'ang'a, Robert Ploutz-Snyder, Rosemary Rochford

**Affiliations:** 1 School of Pure and Applied Science, Kenyatta University, Nairobi, Kenya; 2 Kenya Medical Research Institute, Center for Global Health Research, Kisumu, Kenya; 3 Center for Global Health and Diseases, Case Western Reserve University, Cleveland, OH, USA; 4 Center for Outcomes Research and Evaluation, SUNY Upstate Medical University, Syracuse, NY, USA; 5 Department of Microbiology and Immunology, SUNY Upstate Medical University, Syracuse, NY, USA; 6 Biostatistics Lab, Human Adaptation and Countermeasures Division, National Aeronautics and Space Administration, Houston, TX, USA

## Abstract

**Background:**

The effects of *Plasmodium falciparum *on B-cell homeostasis have not been well characterized. This study investigated whether an episode of acute malaria in young children results in changes in the peripheral B cell phenotype.

**Methods:**

Using flow-cytofluorimetric analysis, the B cell phenotypes found in the peripheral blood of children aged 2–5 years were characterized during an episode of acute uncomplicated clinical malaria and four weeks post-recovery and in healthy age-matched controls.

**Results:**

There was a significant decrease in CD19^+ ^B lymphocytes during acute malaria. Characterization of the CD19^+ ^B cell subsets in the peripheral blood based on expression of IgD and CD38 revealed a significant decrease in the numbers of naive 1 CD38^-^IgD^+ ^B cells while there was an increase in CD38^+^IgD^- ^memory 3 B cells during acute malaria. Further analysis of the peripheral B cell phenotype also identified an expansion of transitional CD10^+^CD19^+ ^B cells in children following an episode of acute malaria with up to 25% of total CD19^+ ^B cell pool residing in this subset.

**Conclusion:**

Children experiencing an episode of acute uncomplicated clinical malaria experienced profound disturbances in B cell homeostasis.

## Background

There are over 500 million episodes of clinical *Plasmodium falciparum *malaria annually [1]. The primary burden of infections with *P. falciparum *occurs mainly in children under five years of age living in the tropical and sub-tropical areas of the world, where malaria transmission is holoendemic [2,3]. Malaria induces many pathophysiological changes including alterations in both T and B cell immunity [4]. In addition, in regions where malaria transmission is holoendemic, immunity is not acquired until after several years of exposure and can be lost rapidly following migration out of a malaria endemic region suggesting poor generation of protective immune memory [5-7]. The mechanism of immune suppression induced by *P. falciparum *remains an important question to solve in order to achieve protective immunity by means of vaccination.

A number of observations clearly indicate that B cells are affected by *P. falciparum *infection. Hypergammaglobulinaemia has been a well-described feature of Plasmodium infections [8] and persons living in malaria holoendemic regions have elevated total antibody levels [9]. Dorfman *et al *[10] reported a diminished frequency of *P. falciparum*-specific memory B cells as indicated by loss of *P. falciparum *antibodies, and Kassa *et al *[11] reported a decline in total numbers of CD19^+ ^B cells. Polyclonal B cell activation induced by *P. falciparum *[9] most likely occurs through antigenic activation by the cysteine-rich interdomain region α (CIDR1α) of *P. falciparum *erythrocyte membrane protein 1(PfEMP1) and merozoite antigens [12].

In addition to disturbances in generation and maintenance of B cell immunity following *P. falciparum *infection, chronic antigenic activation of B cells within the context of repeated *P. falciparum *infections may lead to cytogenic abnormalities or aberrations in B cell development and trafficking. This may be one possible explanation for the increased risk for Burkitt's lymphoma in children living in malaria endemic settings [13].

Although there is some data on the types and frequencies of mature B cells that populate the peripheral blood in healthy children and HIV infected children [14], few studies have examined these changes in B cell subsets as they occur in peripheral blood of children in malaria endemic regions, who bear the burden of malaria related mortality and morbidity. Kassa *et al *[11] found a decrease in total CD19^+ ^B cells following acute *P. falciparum *and *P. vivax *infections, but no further phenotyping of B cell subsets was done. Recent development in B cell biology allow distinctions of peripheral B cells into distinct subsets i.e. naive (CD19^+^IgD^+^) and memory (CD19^+^IgD^-^) subsets based on IgD staining [15]. Further delineation of peripheral B cells was reported by Bonhorst *et al *[15]. They used relative levels on IgD and CD38 expression to identify four different B cell sub-populations in peripheral blood. Other markers to delineate peripheral B cell subsets include CD10, which was initially thought to be a marker for germinal center B cells and Burkitt lymphoma cells [16,17]. However, CD10 was recently shown to be expressed on a subset of peripheral B cells known as immature transitional B cells [18,19]. Infection with HIV was shown to increase CD10^+^CD19^+ ^immature transitional B cells suggesting that the transitional B cells are potential marker of inflammation. Whether *P. falciparum *infection induces these cells is unknown.

To understand how *P. falciparum *infection modulates B cell homeostasis, flow cytofluorimetric (FCF) analysis was used to quantify the percentage of total B cells (CD19^+^) and to discriminate B cell subsets in the peripheral blood in children experiencing an acute clinical case of *P. falciparum *malaria and four weeks following recovery. In this study, it was shown that following acute clinical *P. falciparum *malaria there were significant increases in CD10^+ ^B cells as well as a decline in CD19^+^IgD^-^CD38- population of memory B cells.

## Methods

### Study area

This study was carried out at Chulaimbo Rural Training Center during the months of June and July 2003. This clinic serves a malaria holoendemic area [13] and is located 25 km from Kisumu City in Nyanza Province, Western Kenya. Falciparum malaria accounts for 98% of malarial infections in this region.

### Study participants

All the protocols used in this study were reviewed and approved by the Kenya Medical Research Institute (KEMRI) Ethical Review Committee and Institutional Review Board of Human Studies at University Hospitals of Cleveland, Case Western Reserve University, USA. After informed consent was obtained from the parents or guardians, twenty-five children aged 2–6 years presenting with acute clinical malaria (based on clinical signs determined by a clinical officer) were enrolled in the study. All malaria cases were treated according to the Kenyan national malaria treatment policy set in 1998 which using sulphadoxine-pyrimethamine (SP) as the first-line anti-malaria treatment. Inclusion criteria for children in the study included parental/guardians consent for blood samples to be drawn twice within four weeks, age two to six years, and admission with a diagnosis of acute malaria morbidity as defined by an axillary temperature ≥37.5°C and *P. falciparum *parasitaemia ≥5,000 parasites/μl. Children were excluded from the study if they were severely immunosuppressed, malnourished, or had other concurrent infections that may cause fever such as lower respiratory tract infection; had haemoglobin (Hb) less than 5 g/dl; and parasitaemia with fever at the follow-up visit four weeks after enrollment in this study or relocation from study area. Follow-up was conducted four weeks post-recovery. Fifteen children met the inclusion criteria and were further analysed. Ten children were excluded from the study at follow-up based on the exclusion criteria (e.g. relocation from study area, parasitaemia with fever at the follow-up, and Hb levels needing transfusion between enrollment and follow-up). Eleven healthy-age matched children who were parasitaemic, but asymptomatic, and from the same geographic area were included as controls. The clinical and the demographic data of the study participants are displayed in Table 1.

**Table 1:** General clinical characteristics of the study subject

**Study participant status**	**Acute clinical malaria, n = 15**	**Post recovery, n = 15**	**Controls, n = 11**
Temperature (°C) [range]	37.8* [37.5–39.9]	36.5 [34.9–37.3]	36.3 [35.4–36.8]
Hemoglobin levels (g/dl) [range]	8.6 [6.5–12.4]	10.3 [6.2–14.0]	10.67 [8.6–11.6]
Parasite density/μl [range]	47066 [11280–141960]	3464 [0–18280]	1820 [680–3000]

*Values are arithmetic mean of the variables assessed in the three clinical groups

### Blood collection

After measurements of haemoglobin levels using a portable B-haemoglobin photometer (Hemocue AB Angelholm, Sweden), thick and thin blood films were prepared from finger prick samples and stained with 5% Giemsa to determine *Plasmodium *species and level of parasitaemia. Following microscopic confirmation of *P. falciparum *parasitaemia of sufficient density, 2–5 ml of blood whole blood was collected in EDTA vacutainer by venipuncture.

### Lymphocyte isolation and cryopreservation

Blood was anticoagulated in EDTA and peripheral blood mononuclear cells (PBMC) were separated from whole blood by Ficoll-Hypaque density gradient centrifugation within two hours of venipuncture. Cell viability was determined using the trypan blue dye exclusion assay. Cells were resuspended in 1 ml of freezing media (50% foetal bovine serum, 40% RPMI, and 10% DMSO) and stored at -80°C for later batch analysis by flow cytometry.

### Immunophenotyping of peripheral blood lymphocytes

Cryropreserved peripheral blood mononuclear cells were quickly thawed in a water bath at 37°C, washed in cold PBS with 3% BSA and 10 mM HEPES. PBMC were enumerated using a heamocytometer, and viability routinely assessed to be above 90%. A total of 3 × 10^5 ^cells/100 μl flow buffer (PBS, 3% BSA) were stained on ice with the following antibodies to lymphocyte surface receptors: CD3-FITC, CD8-FITC, IgD-FITC, CD38-FITC, CD19-PE, CD4-PE, CD38-PE, CD-10PE, CD23-PE, CD45-APC, CD3-APC, and CD19-APC (BD Pharmingen, CA, San Diego, USA). Isotype controls were IgG_2a_, K -FITC (mouse), IgG_1_, K -PE (mouse) and IgG_1_, K -FITC (mouse) (BD Pharmingen). After staining, cells were fixed with 1% paraformaldehyde for 15 minutes, and then resuspended in 300 μl flow buffer for analysis on a FACS Calibur flow cytometer (Becton Dickinson Immunocytometry Systems, San Jose, USA). Data was analysed using CellQuestPro software (BD Immunocytometry Systems).

### Immunoglobulin assays

Total immunoglobulin (IgG, IgM and IgA) was measured using standard capture and detection sandwich enzyme linked immunosorbent assay (ELISA) [20]. The plates were read on an automated OpsysMR microplate reader at 410 nm (DYNEX Technologies, USA). Immunoglobulin concentration were determined by extrapolation from the standard curves.

### Statistical analysis

Paired T-test was used to compare the differences in means of the same individuals at different time points (clinical patients, acute versus post-recovery). Independent-measures T-test was used to compare differences between acute clinical patients, post-recovery and an age-matched control group. Heterogeneity of variance assumptions was tested by the Levene statistic. In all cases P < 0.05 were considered statistically significant. SPSS v16 (SPSS, Inc., Chicago, IL) was used for statistical analysis.

## Results

### General characteristics of the study population

Twenty-five children presenting with acute clinical malaria were enrolled in this study. However, during follow-up conducted four weeks post recovery, only fifteen children met enrollment criteria and were included in the subsequent analysis. Clinical characteristics of the study participants are shown in Table 1. The mean age of the enrollees was 42.4 months, while controls were 42.8 months. Children presenting with acute uncomplicated clinical malaria had a mean *P. falciparum *parasite density/μl of 47,066 ± 32,105. Four weeks following treatment for malaria, the parasite density had dropped in all cases with a mean density of 3,464 parasites/μl. Some of the controls were asymptomatic, but parasitaemic and had a mean density of 1,820 parasites/μl. Children who had acute clinical malaria had the highest temperature (mean of 37.8°C), whereas during recovery they had comparable temperature to the control children (mean of 36.5°C). The haemoglobin levels were 8.6 g/dL, 10.3 g/dL, and 10.7 g/dL for acute clinical malaria, post-recovery and controls respectively.

### Changes in CD19^+ ^B cells in children with acute clinical malaria

Since acute clinical malaria has been associated with perturbation of lymphocyte populations [11,21], the frequency of T and B cells in peripheral blood of children was first examined during acute clinical malaria and post-recovery. These values were then compared with T and B cell frequencies in healthy age-matched controls. Following recovery from malaria, children had a significantly higher percentage of CD19^+ ^B cells relative to when they had an episode of acute clinical malaria (P = 0.02) (Table 2). In contrast, no significant differences in CD3^+ ^T cells were observed in the same children during acute versus recovery phases of malaria. To further delineate the T cell subsets, the percentage of CD3^+ ^CD4^+ ^and CD3^+^CD8^+ ^cells in the same samples was analyzed. While no significant differences were observed in the percentages of CD4^+ ^and CD8^+ ^cells between children with acute malaria and following recovery, significantly fewer CD3^+ ^CD4^+ ^cells (P = 0.04) and more CD3^+^CD8^+ ^T cells (P = 0.03) were measured in healthy age-matched controls relative to the children that had recovered from acute malaria.

**Table 2:** Mean percentage of lymphocyte subsets at presentation with acute non-complicated malaria, 4 weeks post-recovery and in healthy age-matched controls

	Acute (A)		Recovery (R)		Controls (C)		Significance		
	
Cell type	mean (SEM) percent		mean (SEM) percent		mean (SEM) percent		A vs.R	A vs.C	R vs.C
CD3^+^CD45^+^	56.9	(2.4)	54.3	(2.3)	52.7	(2.7)	0.37	0.27	0.67
CD19^+^CD45^+^	21.4	(2.1)	26.1	(1.7)	23.5	(2.8)	**0.02**	0.53	0.41
CD4^+^CD3^+^	34.8	(2.6)	36.0	(2.2)	29.4	(1.9)	0.64	0.14	**0.04**
CD8^+^CD3^+^	16.0	(2.5)	12.8	(1.2)	18.2	(0.8)	0.22	0.62	**0.03**

Naive 1 CD38-IgD^+^CD19^+^	23.7	(2.9)	29.3	(4.2)	38.2	(3.4)	**0.03**	**0.00**	0.12
Naive 2 CD38^+^IgD^+^CD19^+^	39.7	(3.8)	44.7	(4.4)	27.2	(4.4)	0.11	**0.04**	**0.01**
Memory 3 CD38^+^IgD^-^CD19^+^	22.7	(2.4)	14.4	(1.3)	14.2	(1.1)	**0.00**	**0.01**	0.91
Memory 4 CD38-IgD-CD19^+^	13.5	(1.4)	11.7	(1.3)	22.8	(2.6)	0.11	**0.01**	**0.00**

CD10^+^CD19^+^	24.6	(2.5)	29.0	(2.7)	21.2	(1.8)	**0.04**	0.31	**0.04**

Sample size (n) was n = 15 for all Acute & Recovery outcomes except CD19^+^CD10^+ ^(n = 12), and all Memory and Naive outcomes (n = 11). N = 11 for controls except for CD4 (n = 10), CD19+CD10^+ ^(n = 9), CD19^+^CD27^+ ^(n = 7), and CD8 (n = 5). Heterogeneity of variance assumptions were tested by the Levene statistic, and heterogeneous t-tests were utilized as needed. Significant differences are in **bold**.

### Alterations in memory and naive B cell subsets in children with acute clinical malaria

**Figure 1:**
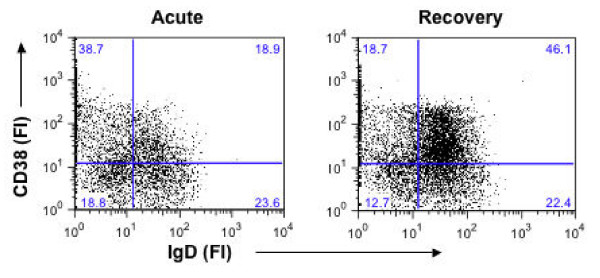
**Alterations in peripheral B cell subsets in children with acute malaria compared to the same children following recovery**. Representative FCF analysis of CD19^+ ^B cells stained for CD38 and IgD. Expression of CD38 and IgD can be used to identify naive and memory B cell subpopulations in the peripheral blood [15].

Four major populations of B cells in the peripheral blood can be distinguished based on expression of IgD and CD38 [15]: naive 1 (CD38^-^IgD^+^), naive 2 (CD38^+^IgD^+^), memory 3 (CD38^+^IgD^-^) and memory 4 (CD38^-^IgD^-^). To determine the frequencies of these B cell subsets in the study population, PBMC were live-gated on CD19^+ ^cells to collect sufficient number of cells for analysis and then analysed for expression of IgD and CD38. A representative FCF analysis is shown in Figure 1A and results for all samples are reported in Table 2. Children with acute clinical malaria had significantly lower naive 1 (CD38^-^IgD^+^) B cell subsets compared to the same population following recovery (P = 0.03) and to age-matched healthy controls (P = 0.00). In contrast, elevation of the memory 3 (CD38^+^IgD^-^) B cell subset was observed in the acute clinical malaria compared to the same population following recovery (P = 0.00) and to age-matched healthy controls (P = 0.01). Interestingly, the memory 4 (CD38^-^IgD^-^) B cell subset was significantly lower in both acute clinical malaria and recovery compared to healthy controls (P = 0.01 and P = 0.00 respectively). This mirrors the increase in the naive 2 (CD38+IgD+) B cell subset during both acute clinical malaria and recovery compared to healthy controls (P = 0.04 and P = 0.01 respectively).

### Falciparum malaria results in increases in transitional CD19^+^CD10^+ ^B cells in peripheral blood

CD10 was originally thought to be expressed primarily by germinal center B cells and Burkitt's lymphoma tumor cells [17]. However, recently CD10 expression has been associated with immature transitional B cells [18,19]. To determine whether acute clinical *P. falciparum *induces increased expression of CD10, phenotypic analysis of peripheral blood of children during acute clinical malaria, following recovery and in asymptomatic children was performed. The results illustrate that following recovery from malaria, children had significantly higher levels of CD10 expression than during acute clinical malaria (P = 0.04) and also compared to controls (P = .04) (Table 2). Importantly, it was confirmed that the peripheral B cells expressing CD10 were negative for CD34 or CD27.

### Acute clinical malaria results in hypergammaglobulinaemia

**Figure 2:**
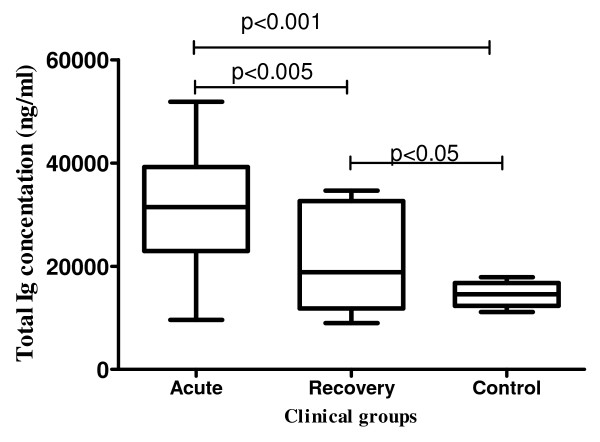
**Comparison of serum Ig levels in children with acute malaria, following recovery and age-matched controls**. Total human Ig (IgG, IgM and IgA) in plasma was determined by ELISA analysis. Shown are mean (SEM) Ig levels between children with acute malaria (n = 15), the same children following recovery and age matched healthy children (n = 6). Significant differences between acute (a), recovery (r), and controls (c) as noted.

Previous studies have demonstrated that acute clinical malaria is associated with hypergammaglobulinaemia [8]. To determine if this was also true for these study participants, quantitative total immunoglobins (Ig) were measured using ELISA in the plasma obtained from PBMC. As shown in Figure 2, increased levels of total Ig were evident during acute clinical malaria, with children having significantly higher Ig levels during acute clinical malaria as compared to post recovery (p < 0.005) and the controls (p < 0.001). Additionally the Ig levels were significantly higher post recovery as compared to controls (p < 0.05).

## Discussion

Although extensive information is available on the types and frequencies of mature B cells that populate the peripheral blood, few studies have been done analysing how B-cell lymphopoiesis is altered in children infected with *P. falciparum *malaria. In this study, a significant decline in the percentages of CD19^+ ^B cells in the peripheral blood of children during an episode of acute clinical *P. falciparum *malaria compared to the same children post-recovery and to healthy age-matched children was observed. In addition, the children with acute malaria had an increase in a memory B cell subset and a concomitant decrease in a naive B cell subset in children as compared to the same children post-recovery and to healthy age-matched children. Thus, the data presented here suggests that *P. falciparum *infection results in perturbations of the B cell compartment and is consistent with accumulating evidence from a number of other studies showing dysregulation of B cell function following *P. falciparum *infection [11,21].

Lymphopaenia has long been recognized as a complication of acute malaria [22]. Consistent with this, there was a decline in percentages of CD19^+ ^B cells during acute malaria in this study. However, there were no significant differences in CD3^+ ^T cells in this same population. Although the immunological pathways that orchestrate B lymphocyte depletion are unknown, *P. falciparum *malaria may interfere with B cell lymphopoiesis, induce lymphocyte apoptosis as shown following infection of macaques with *P. coatneyi *[23], or result in sequestration of lymphocytes to other tissues or endothelium [22]. Lastly, given the hypergammaglobulinaemia that this study and others [8] have observed following *P. falciparum *infection, the possibility exists that *P. falciparum *infection, possibly through direct interactions of *P. falciparum *antigens with memory B cells, is driving memory B cells towards plasma cell differentiation and migration of these cells back to the bone marrow. Consistent with this, changes in B cell subsets were observed, with significantly higher memory 3 B cell subset observed in acute clinical malaria compared to recovery or controls, and a lower percent of memory 4 B cell subset in children both during acute and following recovery compared to healthy age-matched controls. Taken together, these observations have important implications in the maintenance of immunologic memory to vaccine antigens in populations chronically exposed to malaria infections, especially in children since they are most vulnerable to this parasitic infection.

Elevated levels of the IgD^+^CD38^+ ^naive 2 equivalent of pre-germinal center B cells have been reported in the peripheral blood children with systemic lupus erythematosus [24], but this B cell subset is rarely observed in peripheral blood of healthy individuals. This study also reported elevated levels of these pre-germinal center B cells in children following recovery from acute clinical malaria. It is unclear whether this reflects interference of *P. falciparum *infection with B cell trafficking and/or differentiation, which could lead to cells exiting secondary lymphoid organs at early differentiation stages. A recent report found that the architecture of the germinal center was disrupted in a mouse model of malaria [25]. Furthermore, Urban and colleagues also observed disruption of splenic organization in autopsy studies of adults who died of *P. falciparum *malaria [26]. Together, these studies point towards *P. facliparum *infection resulting in alterations of lymphoid architecture, which could result in the changes in peripheral B cell subsets that were observed in this study.

An expansion of CD10^+^CD19^+ ^B cells was observed following recovery from acute clinical *P. falciparum *malaria, with up to 25% of total CD19^+ ^B cell pool residing in this subset. The cells expressing CD10 cells were IgD^+ ^and CD34^-^, suggesting that they were not pre-B cells that had aberrantly migrated to the periphery from the bone marrow [27,28]. Studies in autoimmune and immunodeficiency diseases have associated the expression of CD10 with immature transitional B cells and their elevated levels in peripheral circulation are thought to be indicative of B cell dysfunction [18]. Interestingly, Burkitt lymphoma express CD10 and it this has led to the idea that these tumors may be derived from CD10^+ ^germinal center B cells [17]. It is possible that *P. falciparum *infection induces immature B cells to leave the bone marrow at an earlier differentiation stage [15,19], and thereby increase proliferation of B cell subsets that are precursors of eBL. This may explain the increased risk for Burkitt's lymphoma in children living in malaria holoendemic regions.

The methods for determining the percentage of lymphocyte subsets in peripheral blood have over time, switched from PBMC isolation and then phenotyping to direct phenotyping from whole blood. The phenotype of peripheral blood lymphocytes was analysed following isolation of PBMC because of the difficulties in timing when samples were collected, the desire to do a batch analysis on all the samples to ensure consistency in our analysis, and the inherent challenges of the clinical setting in Kenya in which we were working. There are very few published studies of lymphocyte subsets in healthy children and most are based on children living in developed countries. The present study observed that the mean CD19^+ ^B cells as a percentage of CD45^+ ^lymphocytes was 23.53% in healthy controls. This is within the range reported in healthy children and HIV infected children and is much higher than observed in healthy adults where the range is typically 12% of total lymphocytes [14]. The present study also reported higher levels of CD10^+^CD19^+ ^B cells than reported for one other study examining CD10^+ ^levels in HIV infected children [14].

## Conclusion

In summary, these results demonstrate that during acute uncomplicated episodes of *P. falciparum *malaria in children, there were major perturbations in B cell subsets including a decline in memory B cells and an increase in transitional CD10^+ ^cells. Understanding the mechanisms underlying the alterations in B cell lymphopoeisis are needed to aid in vaccine development.

## Competing interests

The authors declare that they have no competing interests.

## Authors' contributions

ASA carried out the cell isolation, cell analysis and drafted the manuscript. AMM participated in the design and coordination of the study and draft of manuscript. CK participated in recruitment, FCF analysis and draft of manuscript. ZWN participated in study design. RPS carried out the statistical analysis. RR conceived of the study, and participated in its design and coordination and helped to draft the manuscript. All authors read and approved the final manuscript.
